# Overall Survival and Prognostic Factors in Osteosarcoma: A Nationwide Cohort Study from Taiwan

**DOI:** 10.3390/life16020288

**Published:** 2026-02-08

**Authors:** Hsi-Yu Chu, Xiao-Han Shen, Chia-Nan Lin, Chung-Chen Lee, Yong-Chen Chen, Yu-Jen Chen, Yu-Chih Lin, Shu-Hao Chang

**Affiliations:** 1 School of Medicine, College of Medicine, Fu Jen Catholic University, No. 510, Zhongzheng Rd., Xinzhuang District, New Taipei City 24205, Taiwan; charliechu0713@gmail.com; 2 Post-Baccalaureate Program in Nursing, College of Medicine, Fu Jen Catholic University, New Taipei City 24205, Taiwan; a0983700983@gmail.com (X.-H.S.); 137159@mail.fju.edu.tw (Y.-C.C.); 3 Department of Medical Imaging, Fu Jen Catholic University Hospital, Fu Jen Catholic University, New Taipei City 24352, Taiwan; a00411@mail.fjuh.fju.edu.tw; 4 Data Science Center, College of Medicine, Fu Jen Catholic University, No. 510, Zhongzheng Rd., Xinzhuang District, New Taipei City 24205, Taiwan; rainbow2748@gmail.com; 5 Research and Development Center for Physical Education, Health, and Information Technology, Fu Jen Catholic University, New Taipei City 24205, Taiwan; yujenc@gmail.com; 6 Department of Orthopedic Surgery, Chang Gung Memorial Hospital, Linkou Branch, No. 5, Fuxing Street, Guishan District, Taoyuan City 33305, Taiwan; b101092127@cgmh.org.tw; 7 Department of Orthopedics, Fu Jen Catholic University Hospital, Fu Jen Catholic University, No. 69, Guizi Rd., Taishan District, New Taipei City 24352, Taiwan

**Keywords:** osteosarcoma, overall survival, prognostic factor, nationwide cohort

## Abstract

Osteosarcoma is the most common primary malignant bone tumor, predominantly affecting adolescents and young adults. Survival outcomes vary widely, and previous studies have suggested differences in patient characteristics and disease patterns across populations; however, population-based evidence from Taiwan remains limited. In this nationwide retrospective cohort study, data from the Taiwan Cancer Registry were analyzed to evaluate overall survival (OS) and prognostic factors among patients diagnosed between 2013 and 2022. A total of 371 patients were included, with a mean follow-up of 4.6 years. Kaplan–Meier analyses demonstrated significant differences in overall survival according to tumor stage, primary tumor site, and histological grade (all log-rank *p* < 0.05). In multivariable Cox proportional hazards analyses, older age groups were associated with an increased risk of death, while high-stage disease (AJCC stage III–IV) was associated with a significantly higher risk of mortality compared with low-stage disease (HR = 1.94, 95% CI = 1.30–2.90). In contrast, low-grade histology (HR = 0.31, 95% CI = 0.14–0.71) and limb salvage surgery (HR = 0.38, 95% CI = 0.23–0.64) were associated with improved survival. These findings provide population-based evidence on prognostic factors for osteosarcoma in Taiwan and highlight the associations of age, disease stage, tumor biology, and surgical management with overall survival.

## 1. Introduction

Osteosarcoma is the most common primary malignancy of bone which typically occurs in children and the elderly, showing the bimodal distribution of incidence at ages 0–24 and a second peak for age > 65 [1]. As for the situation in Taiwan, osteosarcoma accounts for <0.1% of all cancers, with age-standardized incidence rates (ASRs) of 3.01 per million which is consistent with previous studies worldwide. It also revealed a peak for incidence in children and young adolescents [2]. However, the incidence rate in the elderly was much lower than the world average and the incidence trend of osteosarcoma was significantly rising in Taiwan [2,3]. This result is consistent with findings from another report using data from the Cancer Incidence in Five Continents (CI5), which revealed that Eastern Asia, in contrast to most other regions, demonstrated a statistically significant rising trend in osteosarcoma incidence over time [4]. These findings suggested potential differences in patient characteristics and disease patterns between countries and populations, reinforcing the importance of Taiwan-specific study. As for survival outcomes, a study using the SEER database reported a 5-year cause-specific survival rate of 71.8% for patients without metastasis and 30.4% for those with metastasis at diagnosis [5]. A single institutional report from Taiwan showed a 5-year overall survival (OS) rate of 77% for all patients younger than 18 years old [6]. Later in 2016, a study from the same institution reported OS rates in a broader patient population beyond typical young patients. For all high-grade osteosarcoma cases diagnosed between 2004 and 2011, the 5- and 10-year OS rates were 72.6% and 66.7%, respectively [7]. Additionally, it had been reported that up to 25% of patients present with the sign of metastasis at diagnosis and the OS rate for those with metastasis decreased to approximately 33.9% in the aforementioned study [7,8]. These results revealed that osteosarcoma could result in a wide range of prognoses according to different individual conditions. Since most of the patients were children and young adults, they relied heavily on family support. The disease would not only have great impact on them but also their family. However, there have been no recent reports regarding OS and prognosis for patients with osteosarcoma in Taiwan in recent years. In addition, the data from previous studies were mainly derived from hospital-based studies. Therefore, in this study, data from the Taiwan Cancer Registry (TCR) database was collected in the hope of obtaining the most comprehensive and up-to-date data on osteosarcoma in Taiwan and identifying potential prognostic variables and to provide population-based evidence that may serve as a foundation for the future development of a Taiwan-specific prognostic nomogram.

## 2. Materials and Methods

This was a nationwide, population-based retrospective cohort study conducted using data from the TCR. In the study, we focused on patients diagnosed with osteosarcoma between 2013 and 2022, with the aim of analyzing the OS and identifying prognostic factors.

### 2.1. Data Source and Patient Selection

In this study, the data were collected from the long-form database of the TCR. The TCR is a nationwide system established by the Ministry of Health and Welfare in 1979 to provide complete national data for cancer surveillance and control policies [9]. Since then, the TCR has collected newly diagnosed cancer cases from hospitals with 50 or more beds in Taiwan. Moreover, the TCR implemented a long-form database in 2002 which provided more detailed information on the diagnosis, treatment and recurrence of the cases. To date, 80 hospitals, accounting for over 90% of all cancer cases nationwide, participate in the long-form registration [10]. Moreover, according to the annual report from the TCR in 2022, the completeness of the TCR was 98%, the percentage of morphological verification was 95.2% for all sites and 98.73% for cancer of bone and articular cartilage, and the percentage of cases with a death certificate only was 0.75% [11].

In our study, osteosarcoma was defined based on the International Classification of Diseases for Oncology, third edition (ICD-O-03). Patients coded as ICD topography code C40–41 with morphology codes 91803, 91813, 91823, 91833, 91843, 91853, 91863, 91873, 91923, 91933, 91943, 91953 were included in our study. To capture extra-skeletal osteosarcoma, which is not classified under C40–C41, cases coded as C49.9 with morphology code 91803 were also included to ensure complete case ascertainment.

A total of 398 patients diagnosed with osteosarcoma between 2013 and 2022 were initially identified. Patients with missing tumor stage information (*n* = 27) were excluded from the analysis, as tumor stage is a key prognostic variable. After applying this exclusion criterion, 371 patients were included in the final analytic cohort.

### 2.2. Demographic and Clinical Variables

To identify potential factors associated with overall survival, the following demographic and clinical variables were extracted for eligible patients: year of diagnosis, age at diagnosis, sex, tumor stage, primary tumor site, histological grade, and treatment modalities, including surgery, chemotherapy, and radiotherapy. Age at diagnosis was categorized into clinically relevant groups (<18, 18–64, 65–74, and ≥75 years) for analysis. Tumor size was recorded in centimeters and treated as a continuous variable; cases with missing tumor size information were retained in the cohort and included in descriptive and univariate analyses. No imputation was performed for missing tumor size or other clinical variables.

Primary tumor site was classified as limb (upper or lower extremity) or axial skeleton. Tumors involving overlapping lesions or unspecified primary bone sites that could not be strictly classified as axial or appendicular were grouped into an “Other” category.

Regarding tumor staging, patients diagnosed between 2013 and 2017 were staged according to the 7th edition of the American Joint Committee on Cancer (AJCC) staging system, whereas those diagnosed in 2018 or later were staged using the 8th edition. To ensure comparability across staging editions and to minimize potential misclassification arising from differences in substaging definitions, tumor stage was harmonized into two categories based on disease extent. Low stage was defined as AJCC stage I or II, representing localized disease, and high stage was defined as AJCC stage III or IV, representing regionally advanced or metastatic disease.

Treatment variables (surgery, chemotherapy, and radiotherapy) were recorded as binary indicators (yes/no). Surgical procedures were further categorized as limb salvage surgery, amputation, or surgery not otherwise specified (NOS), based on registry coding.

### 2.3. Statistical Analysis

The primary endpoint of this study was OS, defined as the time from the date of osteosarcoma diagnosis until death from any cause or the end of the observation period (31 December 2023), whichever occurred first. Patients who were still alive at the end of our observation time (31 December 2023) were censored at that date. The deaths of patients were collected by linking to the profile of the National Death Registry which provided comprehensive nationwide death records.

Continuous variables were summarized using medians and interquartile ranges and compared using the Wilcoxon rank-sum test, while categorical variables were compared using the chi-square test or Fisher’s exact test, as appropriate. Overall survival was estimated using the Kaplan–Meier method, and differences between groups were assessed using the log-rank test. To evaluate the associations between clinicopathological variables and overall survival, univariable and multivariable Cox proportional hazards regression models were constructed, and hazard ratios (HRs) with corresponding 95% confidence intervals (CIs) were reported. Variables with clinical relevance and those showing evidence of association in univariable analyses (*p* < 0.10) were considered for inclusion in the multivariable model. The proportional hazards assumption was assessed using Schoenfeld residuals, and the final multivariable model was specified after confirming acceptable proportional hazards assumptions for the included covariates. A two-sided significance level of α = 0.05 was applied for all statistical tests. All statistical analyses were performed using SAS software, version 9.4 (SAS Institute, Cary, NC, USA).

All of the research protocol was approved by the Institutional Review Board of Fu-Jen Catholic University (No. C110216); date of approval: 20 July 2022.

## 3. Results

### 3.1. Patients

**Figure 1:**
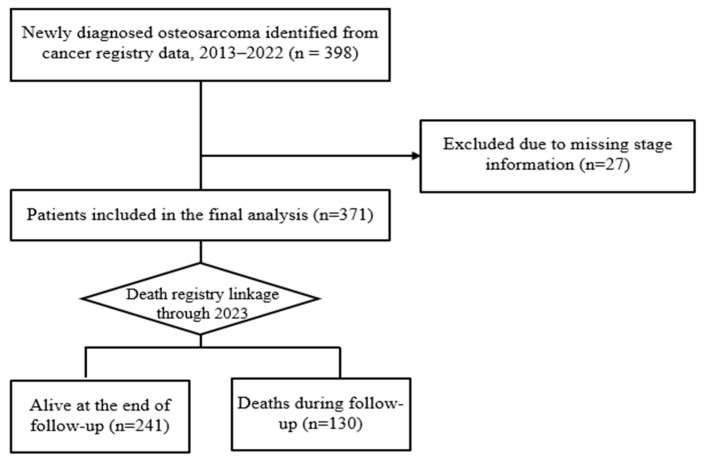
Flow chart for selecting study population.

From 2013 to 2022, a total of 398 patients were newly diagnosed with osteosarcoma in the TCR database. After applying the predefined exclusion criteria (Figure 1), 27 patients were excluded due to missing tumor stage information, and 371 patients were included in the final analysis. The total follow-up time was 1696.4 person-years with a mean follow-up duration of 4.57 ± 2.98 years. During the observation period, 241 (64.96%) were alive and 130 (35.04%) died.

The baseline demographic characteristics of the study population are listed in Table 1. The majority of patients had high-grade osteosarcoma (*n* = 328, 88.41%), and the most common primary tumor site was the lower limb (*n* = 248, 66.85%). Significant differences between survivors and non-survivors were observed across age groups (*p* < 0.0001), tumor stage (*p* = 0.0002), primary site (*p* = 0.0171) histological grade (*p* = 0.0061), surgery status (*p* < 0.0001), and radiotherapy (*p* = 0.0262). On the other hand, there were no statistically significant differences in variables such as sex (*p* = 0.9129), tumor size (*p* = 0.9584) and chemotherapy (*p* = 0.5677).

### 3.2. Survival

**Figure 2:**
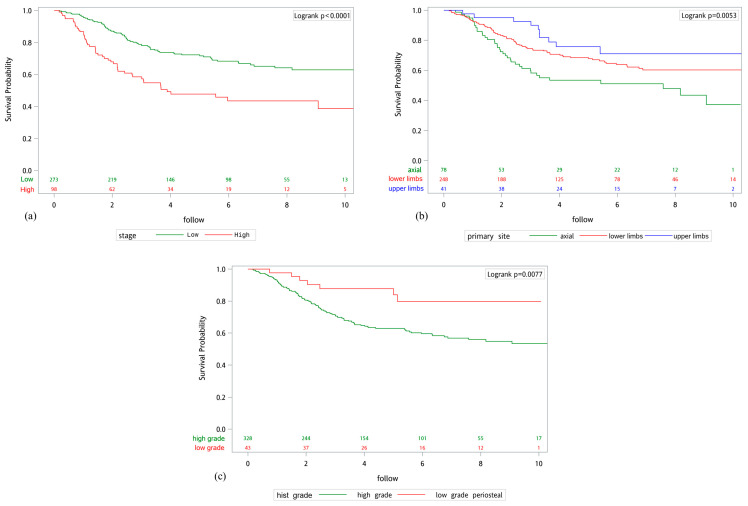
Kaplan–Meier (K-M) curve of patients with Osteosarcoma. (**a**) Stage; (**b**) Primary site; (**c**) Histological grade.

After using a log-rank test in Kaplan–Meier survival analysis, it showed that stage (*p* < 0.0001), primary site (*p* = 0.0053) and histological grade (*p* = 0.0077) were significantly correlated with the OS of the patients (Figure 2). Patients with high-stage osteosarcoma, axial primary tumor site, and high-grade histology demonstrated worse overall survival.

### 3.3. Prognostic Factors

Table 2 shows the results of the univariable and multivariable Cox proportional hazards regression analyses. In univariable analyses, increasing age, advanced tumor stage, axial tumor location, high histological grade, absence of surgery, and receipt of radiotherapy were associated with poorer overall survival, whereas sex, tumor size, and chemotherapy use were not significantly associated with survival.

In the multivariable Cox proportional hazards model, age group, tumor stage, histological grade, and surgical treatment remained independently associated with overall survival. Compared with patients younger than 18 years, mortality risk increased progressively across older age groups, with hazard ratios of 1.91 (95% CI = 1.24–2.93) for patients aged 18–64 years, 3.78 (95% CI = 1.69–8.44) for those aged 65–74 years, and 9.04 (95% CI = 3.67–22.27) for patients aged 75 years or older. Patients with high-stage disease (AJCC stage III–IV) had a significantly higher risk of death compared with those with low-stage disease (HR = 1.94, 95% CI = 1.30–2.90). Low-grade histology was associated with improved survival (HR = 0.31, 95% CI = 0.14–0.71). With regard to surgical treatment, limb salvage surgery was associated with a substantially reduced risk of death compared with no surgical treatment (HR = 0.38, 95% CI = 0.23–0.64). After multivariable adjustment, primary tumor site and radiotherapy were no longer statistically significant.

## 4. Discussion

This nationwide, population-based study provides the most comprehensive assessment to date of overall survival and prognostic factors among patients with osteosarcoma in Taiwan using high-quality registry data from 2013 to 2022. While prior cohort studies and clinical trials have identified factors such as age, tumor site, tumor size, stage, and chemotherapy response as prognostic determinants of overall survival (OS), findings across studies have remained heterogeneous [12,13]. Our analysis contributes new evidence from a large, real-world Taiwanese population and demonstrates that age, tumor stage, histological grade, and surgical treatment are independently associated with OS. In contrast, several factors that appeared significant in univariable analyses did not retain independent prognostic significance after multivariable adjustment.

### 4.1. Age

Age was a significant prognostic factor in our cohort, with older patients showing poorer OS than younger patients. This finding is consistent with a previous meta-analysis and systematic review by Xin et al. [14], which reported similar results. This association may be explained by differences in tumor characteristics among younger patients and their greater ability to tolerate high-dose chemotherapy [15]. Moreover, previous studies have suggested that older patients were more likely to experience delayed diagnosis, face greater surgical challenges, and have a higher likelihood of presenting with metastasis at diagnosis [16]. Together, these factors may contribute to the poorer prognosis observed in elderly patients. In contrast, a retrospective cohort study by Bielack et al. [17] reported that age was a significant prognostic factor in univariate Cox regression analysis but the significance disappeared after multivariate adjustment. The authors attributed the result to potential confounding by other variables such as unfavorable tumor site. This result may be due to relatively fewer elderly patients included in the study since their study mainly focused on children and young adults, which may have limited the ability to detect age-related differences in survival.

### 4.2. Tumor Site

In univariate analysis, axial tumor location was associated with poorer survival, consistent with multiple prior studies. Bielack et al. [17] analyzed patients primarily enrolled in the Cooperative Osteosarcoma Study Group, in which most individuals were younger than 40 years and received standardized, protocol-based treatment. In contrast, Duchman et al. [5] utilized the SEER database, which represented a broader and more heterogeneous real-world population with less uniform treatment information. Despite these differences, both studies consistently demonstrated poorer survival outcomes for patients with axial tumors compared with those with extremity tumors. Specifically, Bielack et al. reported 10-year OS rates of 29.2% for axial tumors and 61.7% for extremity tumors (*p* < 0.0001), while Duchman et al. reported 10-year cause-specific survival rates of 27.0% and 61.8% for axial and extremity sites, respectively (*p* < 0.001). Moreover, both studies identified axial tumor location as an independent risk factor for survival in osteosarcoma (RR = 1.87, 95% CI = 1.25–1.80; HR = 1.85, 95% CI = 1.56–2.19).

In our cohort from the TCR database, which similarly reflects a real-world, nationwide population, we also observed a trend toward poorer survival among patients with axial tumors in the univariate analysis. This may be explained by the fact that axial tumors are more frequently associated with metastatic disease and tend to be larger in size, which makes achieving complete surgical remission more difficult [17,18]. However, in the multivariable analysis, the prognostic significance of tumor site was attenuated. This attenuation may partly reflect the close clinical correlation between tumor site, disease stage, and treatment-related variables, which can reduce the apparent independent contribution of tumor site in multivariable models even in the absence of problematic multicollinearity.

### 4.3. Tumor Grade

Low-grade osteosarcoma was a significant prognostic factor in our study, which is consistent with previous research from Fu et al. [19]. In their retrospective cohort study using data from the SEER database between 2010 and 2016, patients with higher pathological grade were shown to have worse prognoses. Tumor grade refers to the degree of cellular differentiation and the resemblance of tumor cells to normal tissue when observed under a microscope. It reflects how aggressively the tumor is likely to grow and spread and therefore serves as an important prognostic indication. A low-grade tumor is associated with a lower likelihood of metastasis [17,20] which may contribute to the overall survival outcomes observed in these patients.

### 4.4. Surgical Treatment

Limb salvage surgery generally results in better functional outcomes, and advances in surgical technique have further expanded the feasibility of limb salvage, allowing it to be applied in the majority of patients with localized disease. It is now widely accepted that limb salvage is the preferred treatment option in localized osteosarcoma, and amputation typically reserved for lesions that are technically more challenging or not amenable to limb preservation. In our study, patients who underwent limb salvage surgery had a lower risk of death compared to those who did not receive any surgical intervention. These findings suggest that surgical intervention is an important factor associated with OS in osteosarcoma, and limb salvage was associated with the most favorable outcomes in our cohort.

This result is similar to those from Xin et al. [14] They reported a superior result in survival for patients who underwent surgical treatment compared with those who did not receive surgery (HR = 0.45, 95% CI = 0.36–0.57). While most previous studies compared limb salvage surgery with amputation, they still revealed comparable trends showing that limb salvage had better prognosis. Patients selected for surgery do not represent a random subset of all patients with osteosarcoma. Those who undergo amputation or complex procedures for axial or large tumors are often older, have more advanced disease, and present more frequently with metastases and comorbidities, all of which are strong predictors of poor survival. This non-random allocation of treatment reflects indication bias and may explain why, in univariate analyses, some surgically treated subgroups appear to have worse outcomes. After adjusting for tumor stage, grade, and other prognostic variables, the apparent disadvantage associated with these surgical strategies was attenuated, suggesting that the underlying disease severity rather than the surgical method itself accounts for much of the observed difference in survival.

In another retrospective cohort study by Evans et al. [21], they used data from the National Cancer Database (NCDB) to analyze a total of 2442 patients. It showed that limb salvage was associated with superior OS compared with amputation after adjustment and propensity score matching (HR = 0.71, 95% CI = 0.59–0.85). Similarly, a meta-analysis of 17 retrospective studies from Li et al. [22] found no significant difference in local recurrence between limb salvage and amputation (OR 1.03, 95% CI 0.65–1.64) but demonstrated significantly higher 5-year OS in the limb salvage group, with pooled survival rates of 58.60% versus 49.84% for amputation, corresponding to a significant survival advantage (OR = 1.47, 95% CI = 1.10–1.97). Furthermore, limb salvage was associated with a lower incidence of metastasis (OR = 0.24, 95% CI = 0.10–0.60).

Although patients who underwent amputation appeared to have better survival outcomes compared with those who did not undergo surgery, the difference was not statistically significant. The absence of a significant difference in our analysis may be attributed to treatment selection bias (indication bias) and limited statistical power. The number of patients who underwent amputation was relatively small. Moreover, these patients were typically older, had larger and more advanced tumors, were more likely to present with metastases, and had a greater burden of comorbidities and lower socioeconomic status [21]. All of these factors could negatively influence survival outcomes and might therefore offset potential survival benefits of surgery.

### 4.5. Radiotherapy

In our study, radiotherapy was associated with poorer overall survival in univariate analysis; however, this association was no longer significant after multivariable adjustment. Osteosarcoma is traditionally considered a relatively radioresistant tumor, and radiotherapy is not part of standard curative treatment. Instead, it is typically reserved for patients with unresectable tumors, axial lesions, positive surgical margins, or palliative intent in advanced or metastatic disease [8]. Consequently, patients receiving radiotherapy often represent a subgroup with more aggressive tumor biology and limited surgical options, which may explain the inferior survival observed in univariate analysis. After further adjusting other variables in multivariate analysis, the apparent survival disadvantage was then attenuated, suggesting the observed effect reflects underlying disease severity rather than a direct effect of radiotherapy itself.

### 4.6. Chemotherapy

Although chemotherapy is the standard of care for high-grade osteosarcoma, our analysis did not demonstrate a statistically significant survival benefit. This might be attributable to the lack of data on histological response in the TCR database. Previous studies, such as those by Bielack et al. [17] and Miwa et al. [23], have established that histological response is one of the strongest prognostic factors; patients with good histological response have significantly better survival outcomes compared to poor responders. Analyzing the effect of chemotherapy without accounting for histological response may obscure its true therapeutic value. In addition, although more than 80% of patients in the present cohort received chemotherapy, treatment sequencing (e.g., neoadjuvant versus adjuvant chemotherapy) was not incorporated into the multivariable analysis. Furthermore, detailed information on chemotherapy regimens, dosing, and clinical intent was not available. Consequently, chemotherapy was modeled as a binary exposure, and potential heterogeneity in treatment strategies could not be fully captured. Therefore, chemotherapy-related findings in this study should be interpreted with caution.

### 4.7. Limitations and Strengths

While we analyzed patients diagnosed with osteosarcoma from 2013–2022 to completely assess the OS and potential prognostic factors in Taiwan, there are still some limitations that need to be acknowledged. Although the TCR provides high-quality, nationwide data with standardized collection, residual confounding cannot be entirely excluded, especially since some clinical decisions may reflect physician judgment or patient condition, factors that are difficult to capture in registry data. In particular, treatment selection in osteosarcoma is closely linked to tumor severity and patient condition, so some degree of indication bias may remain even after multivariable adjustment.

Second, treatment information in the Taiwan Cancer Registry is recorded as receipt or non-receipt of initial modalities, and treatments were therefore modeled as binary variables rather than as time-dependent covariates. As a result, the potential for immortal time bias cannot be completely excluded. Nevertheless, most patients in our cohort initiated treatment within a relatively short interval after diagnosis, and the primary analyses focused on long-term overall survival, which may mitigate the magnitude of this bias. In addition, given the adjustment for major prognostic variables and the standardized nature of TCR data, the residual indication-related bias is expected to be minimal and unlikely to meaningfully influence our conclusions.

Third, tumor staging was performed using different AJCC editions during the study period. To ensure comparability across staging systems, tumor stage was harmonized into broad categories based on disease extent (stage I–II vs. stage III–IV). Additional analyses demonstrated no significant survival differences between AJCC editions within these harmonized stage groups, suggesting that the staging transition is unlikely to have materially influenced our findings.

In addition, detailed information regarding histological response to chemotherapy was not available for analysis, and thus we were unable to evaluate the impact of treatment response. Finally, lymph node status was unavailable because lymph node dissection is not routinely performed in osteosarcoma. Consequently, we were unable to examine its prognostic role; however, given that current evidence does not consistently support lymph node involvement as a significant prognostic factor in osteosarcoma, this limitation is unlikely to compromise the validity of our conclusions.

Despite these limitations, our study has several notable strengths. This is a retrospective database review, implying the quality of resources may significantly affect the results. Our study is based on the TCR database, which is regarded as a high-quality and comprehensive database, thereby enhancing the reliability of our analysis. To our knowledge, this is the first and largest nationwide, population-based analysis of osteosarcoma survival and prognostic factors in Taiwan. Compared with previous studies, our study may better reflect patient characteristics and treatment outcomes across the entire country.

## 5. Conclusions

In conclusion, this nationwide population-based study analyzed data from patients diagnosed with osteosarcoma in Taiwan between 2013 and 2022 and identified age, tumor stage, histological grade, and surgical treatment as independent prognostic factors for overall survival. The associations of tumor stage and histological grade with survival underscore the importance of early detection and timely diagnosis, which may allow patients to receive treatment before disease progression. Surgical treatment was also identified as an important factor associated with overall survival, highlighting the potential impact of appropriate, guideline-based management in eligible patients. Together, these findings provide population-based evidence on prognostic factors for osteosarcoma in Taiwan and may contribute to a better understanding of prognostic factors and support future clinical research and risk assessment.

## Figures and Tables

**Table 1:** Baseline characteristics of the study population.

Characteristics	Total	Survival *n* (%)/	Death *n* (%)/	*p* Value
		Median (IQR)	Median (IQR)	
All	371	241 (64.96%)	130 (35.04%)	
Age (continuous)	371	18.20 (13.16–30.52)	27.27(15.12–53.86)	<0.0001
Age				
<18	159	119 (74.84%)	40 (25.16%)	<0.0001
18–64	184	112 (60.87%)	72 (39.13%)	
65–74	19	9 (47.37%)	10 (52.63%)	
≥75	9	1 (11.11%)	8 (88.89%)	
Sex				
Male	205	134 (65.37%)	71 (34.63%)	0.9129
Female	166	107 (64.46%)	59 (35.54%)	
Size (cm, continuous) ^#^	311	9.45 (6.60–13.00)	9.00 (6.70–12.50)	0.9584
Stage				
Low stage	273	193 (70.70%)	80 (29.30%)	0.0002
High stage	98	48 (48.98%)	50 (51.02%)	
Primary site				
Upper limb	41	31 (75.61%)	10 (24.39%)	0.0171
Lower limb	248	168 (67.74%)	80 (32.26%)	
Axial	78	40 (51.28%)	38 (48.72%)	
Other *	4	2 (50.00%)	2 (50.00%)	
Histological grade				
Low grade	43	36 (83.72%)	7 (16.28%)	0.0061
High grade	328	205 (62.50%)	123 (37.50%)	
Surgery				
No surgery	31	9 (29.03%)	22 (70.97%)	<0.0001
Limb salvage	331	226 (68.28%)	105 (31.72%)	
Amputation	8	5 (62.50%)	3 (37.50%)	
NOS ^◎^	1	1 (100.00%)		
Radiotherapy				
No	328	220 (67.07%)	108 (32.93%)	0.0262
Yes	43	21 (48.84%)	22 (51.16%)	
Chemotherapy				
No	65	40 (61.54%)	25 (38.46%)	0.5677
Yes	306	201 (65.69%)	105 (34.31%)	

* “Other” category includes overlapping lesions or unspecified sites that could not be strictly classified as axial or appendicular. ^#^ Size missing rate: 16.17%. ^◎^ Not otherwise specified.

**Table 2:** Univariate and multivariate Cox proportional hazards regression analysis of risk factors for patients with osteosarcoma.

	Univariate			Multivariate		
Variables	HR	95% CI	*p* Value	HR	95% CI	*p* Value
Age (continuous)	1.027	1.019–1.036	<0.0001			
Age						
<18	Reference			Reference		
18–64	1.672	1.136–2.460	0.0092	1.909	1.244–2.929	0.0031
65–74	3.918	1.949–7.875	<0.0001	3.779	1.692–8.440	0.0012
≥75	10.839	5.051–23.259	<0.0001	9.036	3.667–22.267	<0.0001
Sex						
Male	0.983	0.696–1.388	0.9219	1.064	0.739–1.532	0.7384
Female	Reference			Reference		
Size (continuous)	1.000	0.996–1.004	0.9551			
Stage						
Low stage	Reference			Reference		
High stage	2.256	1.583–3.214	<0.0001	1.944	1.304–2.898	0.0011
Primary site						
Upper limb	0.644	0.334–1.243	0.1894	0.551	0.282–1.077	0.0813
Lower limb	Reference			Reference		
Axial	1.669	1.134–2.456	0.0094	0.806	0.489–1.328	0.3971
Histological grade						
Low grade	0.370	0.173–0.793	0.0106	0.311	0.136–0.713	0.0058
High grade	Reference			Reference		
Surgery						
No surgery	Reference			Reference		
Limb salvage	0.266	0.168–0.422	<0.0001	0.380	0.228–0.636	0.0002
Amputation	0.367	0.110–1.229	0.1040	0.525	0.149–1.847	0.3154
Radiotherapy						
No	Reference			Reference		
Yes	2.121	1.338–3.361	0.0014	1.396	0.793–2.456	0.2475
Chemotherapy						
No	Reference			Reference		
Yes	0.830	0.536–1.284	0.4018	0.716	0.423–1.210	0.2123
Treatment group						
Surgery + radiotherapy	Reference					
Surgery only	0.535	0.310–0.924	0.0248			
Radiotherapy only	2.933	1.193–7.209	0.0190			
No surgery and no radiotherapy	1.903	0.929–3.896	0.0785			

## Data Availability

The data used in this study are not publicly available due to legal and ethical restrictions imposed by the Health and Welfare Data Science Center, Ministry of Health and Welfare, Taiwan. Access to the data may be granted through an application process administered by the Health and Welfare Data Science Center.
